# Intravenous Vitamin C Administration Improved Blood Cell Counts and Health-Related Quality of Life of Patient with History of Relapsed Acute Myeloid Leukaemia

**DOI:** 10.3390/antiox7070092

**Published:** 2018-07-16

**Authors:** Mike N. Foster, Anitra C. Carr, Alina Antony, Selene Peng, Mike G. Fitzpatrick

**Affiliations:** 1 Integrated Health Options Ltd., Auckland 1050, New Zealand; 2 Department of Pathology and Biomedical Science, University of Otago, Christchurch 8011, New Zealand; anitra.carr@otago.ac.nz; 3 Feedback Research Ltd., Auckland 1050, New Zealand; alina@feedbackresearch.co.nz (A.A.); selene@feedbackresearch.co.nz (S.P.); mike@feedbackresearch.co.nz (M.G.F.)

**Keywords:** vitamin C, intravenous vitamin C, leukemia, acute myeloid leukemia, AML

## Abstract

A 52-year-old female presented to Integrated Health Options Clinic in October 2014 with a history of relapsed acute myeloid leukaemia (AML, diagnosed in 2009 and relapsed in 2014). Intravenous(IV) vitamin C therapy was initiated (in 2014) following completion of chemotherapy as an alternative to haematopoietic stem cell transplantation. IV vitamin C was administered twice weekly at a dose of 70 g/infusion. Within 4 weeks of initiation of IV vitamin C therapy, there was a dramatic improvement in the patient’s blood indices with platelet cell counts increasing from 25 × 10^9^/L to 196 × 10^9^/L and white blood cell counts increasing from 0.29 × 10^9^/L to 4.0 × 10^9^/L, with further improvements observed over the next 18 months. Furthermore, there was a clear and sustained improvement in the patient’s health-related quality of life scores assessed using a validated questionnaire. She has remained healthy and in complete remission until the present day. This case study highlights the benefits of IV vitamin C as a supportive therapy for previously relapsed AML.

## 1. Introduction

Up to 70% of acute myeloid leukaemia (AML) patients treated with chemotherapy will relapse and overall five year survival is about 25% [1,2]. Treatment options for relapsed AML are currently limited to haematopoietic stem cell (HSC) transplantation [3]. Two recent studies have indicated a role for vitamin C in suppressing leukaemia progression through epigenetic mechanisms [4,5]. Vitamin C was found to enhance HSC differentiation and decrease the number of circulating blast cells and leukaemia progression in animal models resulting in improved survival. Animals with vitamin C deficiency were found to have higher levels of HSCs with enhanced self-renewal function, which increases susceptibility to leukaemia. Patients with haematological malignancies have lower vitamin C status than control subjects, with up to 58% exhibiting deficiency [6,7], and further depletion of vitamin C can occur following chemotherapy and HSC transplantation [8,9]. Thus, administration of vitamin C to deficient patients may help to restore normal HSC function and differentiation. Herein we report on a case of relapsed AML who underwent regular IV vitamin C administration instead of HSC transplantation.

## 2. Case Report

The patient signed a consent form allowing the presentation of her case. On 5 May 2009, a 47-year-old female patient was admitted to Auckland Hospital, New Zealand, and diagnosed with Acute Myeloid Leukaemia (AML, bone marrow 75% blasts, normal karyotype, NPM+, FLT3+ with allelic ratio 0.01). She was prescribed daunorubicin/cytarabine (ARA-C) induction, and a Groshong line was inserted. She underwent four cycles of chemotherapy with DA (daunorubicin, cytarabine) × 2MACE (amsacrine, cytarabine, etoposide) and MidAC (mitoxantrone, cytarabine). This was complicated by a presumed Aspergillus invasive pulmonary infection and was treated with oral Voriconazole for three months. A goitre was noted on admission and she was diagnosed with hypothyroidism (thyroid-stimulating hormone (TSH) 54 mU/L, thyroxine (T4) 7.7 pmol/L). She had positive thyroid antibodies and was prescribed thyroxine 50 mcg/day.

Following every cycle of chemotherapy, she was readmitted with a neutropenic fever, which required antibiotic therapy. On 9 May 2009, she developed a neutropenic fever and was started on empiric cefepime and gentamycin while awaiting blood culture results. On those antibiotics, she developed diarrhoea and a rash on her legs, which was diagnosed as Erythema multiforme and thought to be caused by either the cefepime or allopurinol, which she had also been prescribed. Both drugs were discontinued, and she was trialled on meropenem. This drug is a beta-lactam antibiotic given by IV for more serious infections.

By 14 May 2009, her fever persisted and no obvious cause was found at that time. High-resolution computed tomography (HRCT) confirmed that the chest was normal. IV Amphotericin B was commenced. Finally, Staphylococcus epidermidis was grown from a blood culture sample taken from the Groshong line. Amphotericin B was discontinued and IV vancomycin commenced. Her fever was resolved and she was discharged home with IV vancomycin for a further five days. Her platelet count prior to discharge was <10 × 10^9^/L. She was given a platelet transfusion prior to her discharge.

On 25 May 2009, she was readmitted with neutropenic fever. She was again treated with meropenem and vancomycin. She had haemoglobin (Hb) 88 g/L and platelets 20 × 10^9^/L on admission. She was transfused with red blood cells and platelets but developed a reaction to the platelet transfusion, which was managed with hydrocortisone and cetirizine. After two days she was discharged on augmentin for five days and a further two days of vancomycin. The four cycles of chemotherapy were completed by October 2009.

She underwent regular review by the Hematology Clinic until 5 May 2014 when she was discharged from the clinic in complete remission. She remained healthy until August 2014 when she again developed a neutropenia and thrombocytopenia. A bone marrow aspirate on 20 August 2014 revealed a relapsed AML with 61% blasts of similar morphology and cell marker expression as the initial diagnosis. At this time she also had borderline glucose intolerance with HbA1c of 40 mmol/mol. She saw a haematologist, who suggested more intensive chemotherapy, and allogeneic bone marrow transplant. The Haematologist stated, “without a transplant, cure is extremely unlikely and even with a transplant, she is faced with significant transplant related morbidity and an appreciable relapse risk, but with a potential cure opportunity”.

The patient decided to undergo further chemotherapy but rejected a bone marrow transplant. Induction chemotherapy was commenced on 29 August 2014. Bone marrow biopsy on 1 October 2014, showed complete remission of her AML (with 4% blasts). She had no further chemotherapy.

### Intravenous Vitamin C Therapy

On the 10 October 2014, the patient had her first consultation at Integrated Health Options clinic, Auckland, New Zealand. Her Hb at that visit was 93 g/L and platelets 27 × 10^9^/L. There was a discussion with the patient, in the presence of her daughter, about the clinic’s protocol that IV vitamin C infusions are not routinely offered to people with a platelet count of less than 50 × 10^9^/L. After informed consent, she chose to have the IV vitamin C infusions. Her G6PD was normal (tested due to potential haemolytic anaemia with G6PD deficiency). She commenced IV vitamin C therapy on the 7 November 2014 and was prescribed twice weekly IV vitamin C infusions at 70 g/infusion. Her initial pre-infusion plasma vitamin C concentration, measured prior to her first test dose of IV vitamin C on 7 November 2014, was 0.66 mg/dL (37.5 µmol/L). This plasma concentration is considered inadequate, with ≥50 µmol/L (0.88 mg/dL) being adequate and ≥70 µmol/L (1.23 mg/dL) being saturating [10,11]. Post-infusion plasma vitamin C levels were regularly assessed to confirm that the proposed ascorbic acid therapeutic level was achieved (Table 1) [12]. The patient underwent weekly complete blood counts (CBC) and renal function tests, and was also prescribed additional oral supplements (Table 2).

**Figure 1:**
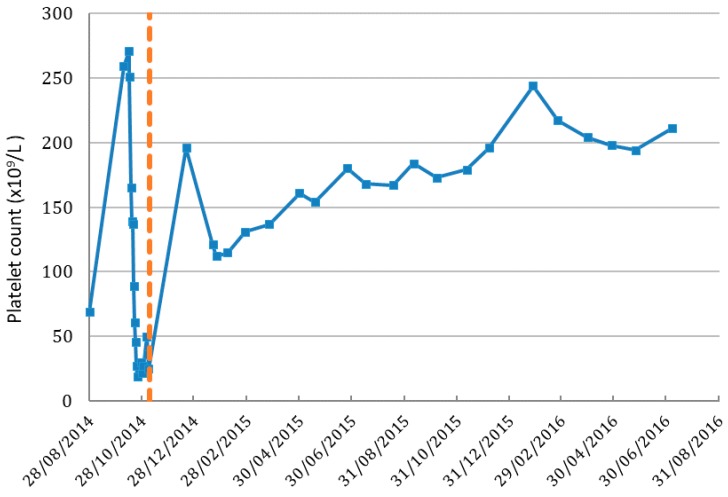
Platelet cell count in patient before and after IV vitamin C treatment. Dashed line indicates the IV vitamin C commenced date. Normal range of platelet count is 150–400 (×10^9^/L).

**Figure 2:**
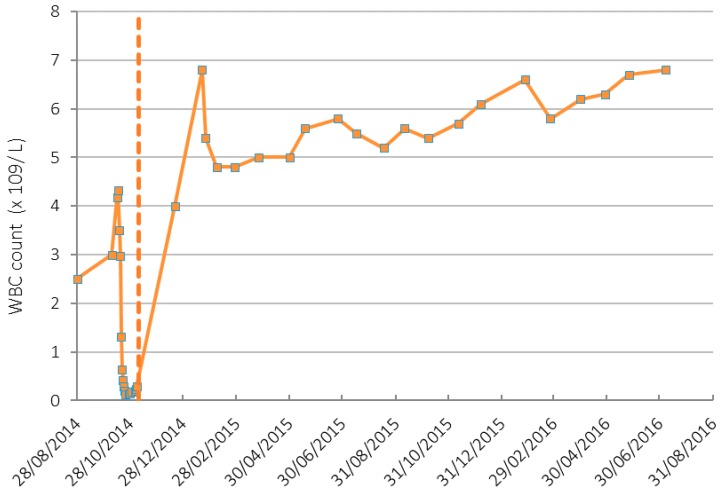
White blood cell (WBC) count in patient before and after IV vitamin C treatment. Dashed line indicates the IV vitamin C commenced date. Normal range of WBC count is 4.5–11 (×10^9^/L).

Prior to initiation of IV vitamin C treatments, the patient’s Hb was 107 g/L, platelets 25 × 10^9^/L and white blood cells (WBC) 0.29 × 10^9^/L. By the 19 December 2014, 42 days after beginning IV vitamin C infusions, her Hb had increased to 111 g/L, platelets to 196 × 10^9^/L (Figure 1) and WBC to 4 × 10^9^/L (Figure 2). On the 7 September 2017 her Hb was 124 g/L, platelet counts were 227 × 10^9^/L, and WBC count was 6.5 × 10^9^/L with a normal differential.

The quality of life (QoL) of the patient was measured before and throughout the IV vitamin C therapy using the validated European Organisation for Research and Treatment of Cancer Quality of Life Questionnaire (EORTC QLQ-C30), which has been used previously to assess QoL following IV vitamin C therapy [16,17]. Six weeks after starting IV vitamin C infusions, her global health status score had increased from 58 to 83, her overall functional score had increased from 89 to 98, and her overall symptom score had dropped from 39 to 8 (Table 3). This was a marked improvement of her QoL, which has been maintained until the present time.

The patient continued twice weekly IV vitamin C infusions of 70 g until August 2015, when the patient felt so well that she decided to reduce the frequency of her IV vitamin C infusions to once weekly. As of 21 September 2017, she has had 141 IV vitamin C infusions. Her blood vitamin C level (AATL) post-infusion averaged at 360 mg/dL (targeted therapeutic range is 350–400 mg/dL) [14,15]. She has now cut back to a monthly infusion but maintains her oral intake of vitamin C as well as the other supplements. The patient remains clinically very well and has remained in complete remission.

## 3. Discussion

The use of complementary and alternative medicine is substantial in the general population [18]. High-dose IV vitamin C, in particular, is sought after by many cancer patients [19]. The rationale for IV administration is the ability to bypass the regulated intestinal uptake of oral vitamin C, thus resulting in 20-fold higher plasma concentrations with IV infusion [20]. These high concentrations of vitamin C are able to indirectly generate hydrogen peroxide through a transition metal ion-dependent mechanism, and this is thought to specifically target and kill cancer cells [21]. IV administration may also be required to facilitate diffusion of vitamin C into tumours with subsequent modulation of important cell signalling pathways [22,23]. Although evidence for the efficacy of vitamin C against solid tumours is currently limited, recent research is indicating a possible role for vitamin C in modulating certain haematological malignancies that are sensitive to the epigenetic regulating effects of vitamin C [4,5]. Vitamin C is able to upregulate the activity of specific tumour suppressor epigenetic enzymes (TETs), which contain mutations in a proportion of AML patients [24,25].

The purpose of this case study was to document the benefits of IV vitamin C as a supportive therapy for previously relapsed AML. In this case, after IV vitamin C treatment, the patient regained normal values for absolute circulating neutrophil count (>1 × 10^9^/L) and platelets count (>100 × 10^9^/L). Cell culture studies have indicated that vitamin C can restore normal tumour suppressor expression profiles and the maturation of HSCs [4,26]. Although the epigenetic (TET mutation) status of the patient was not assessed, it is possible that she had the mutations which respond to vitamin C treatment. The patient remains clinically very well. In addition to vitamin supplements, the patient also incorporated a healthy lifestyle and dietary changes, such as vegetarian diet, yoga, meditation, homeopathic remedies, and herbal remedies (e.g., mistletoe, and black cumin seed oil). These supplements and lifestyle changes may have contributed to the dramatic results achieved by this patient. Therefore, the relative contribution of IV vitamin C therapy could not be singled out. It is also possible that vitamin C may work synergistically with other supplements. Thus, in this case, it was observed that chemotherapy followed by IV vitamin C therapy, combined with a healthy diet and other supplements, supported ongoing patient quality of life and remission of AML.

Administration of chemotherapeutic drugs to patients can result in depletion of vitamin C levels and negatively impact on the patient’s health-related quality of life [17]. Thus, administration of vitamin C to these patients may contribute to improved quality of life through replenishing the essential nutrient and counteracting the toxic side effects of chemotherapy. Indeed, the patient had an inadequate vitamin C status initially, and we observed a marked improvement in the patient’s health-related quality of life following initiation of IV vitamin C therapy, which has been maintained until the present time. The incidence of AML increases dramatically with age and prognosis for the elderly is poor with only 10% surviving five years after diagnosis [2]. As a result of the toxic effects of therapy, including myelosuppression and an increased risk of infection, chemotherapy may not be offered to the very elderly, in which case IV vitamin C may be a viable palliative option for these patients due to its relative safety. Furthermore, a recent trial of low gram dose IV vitamin C administered with DCAG (decitabine, granulocyte colony stimulating factor, cytarabine, aclarubicin) in elderly acute myeloid leukaemia patients showed an increased incidence of complete remission after first induction and prolonged overall survival compared with DCAG alone [27].

## 4. Conclusions

Administration of high-dose IV vitamin C to previously relapsed AML patients may help to restore normal blood cell counts and function. Improved quality of life is also observed. Future clinical trials of IV vitamin C for haematological cancers should target patients with the relevant (TET) mutations, or endeavour to include sub-group analysis of patients with these mutations.

## Figures and Tables

**Table 1:** Intravenous vitamin C dosage infused into the patient and post-infusion ascorbic acid therapeutic level in blood plasma.

Date (Day/Month/Year)	IV Vitamin C Dose (g)	AATL (mg/dL) ^1^	AATL (mmol/L)
21/11/14	70	451	26
8/01/15	70	286	16
9/01/15	75	435	25
17/02/15	70	373	22
24/03/15	70	394	22
8/05/15	70	465	26
12/06/15	65	373	21
17/08/15	65	350	20
15/09/15	65	347	20
1/10/15	65	368	21
3/12/15	65	369	21
9/02/16	65	315	18
19/02/16	65	389	22
24/06/16	65	352	20

^1^ Ascorbic acid was measured using high performance liquid chromatography (HPLC) with electrochemical detection [13]. Targeted level for ascorbic acid therapeutic level (AATL) is 350–400 mg/dL (or 20–23 mmol/L) [14,15].

**Table 2:** Supplementary medication taken concurrently with IV vitamin C infusion by patient.

Supplements	Intake/Day
Alpha lipoic acid ^1^	600 mg
Sodium ascorbate	2000 mg
Methylated B vitamins ^2^	1000 mg
Vitamin D	5000 units
Vitamin K1	1500 mcg
Vitamin K2-MK4	1000 mcg
Vitamin K2-MK7	200 mcg

^1^ Mixture of R and S enantiomers. ^2^ The biologically active form of multiple B vitamins.

**Table 3:** Quality of life score measured using the EORTC QLQ-C30.

Date (Day/Month/Year)	Global Health Status	Functional Scales	Symptom Scales
Baseline (10/10/14) ^1^	58.3	88.9	38.5
6 weeks (17/12/14)	83.3	97.6	7.7
3 months (05/02/15)	83.3	97.8	5.1
6 months (07/05/15)	83.3	95.6	7.7
12 months (11/11/15)	83.3	97.8	7.7

^1^ Baseline survey was completed at first consultation. The following survey rounds were calculated from the first IV vitamin C treatment date—7/11/14.
